# A Higher Serum Anion Gap Is Associated with the Risk of Progressing to Impaired Fasting Glucose and Diabetes

**DOI:** 10.1155/2021/4350418

**Published:** 2021-12-13

**Authors:** Yingchao Zhang, Fengran Xiong, Ruxuan Zhao, Tingting Shi, Jing Lu, Jinkui Yang

**Affiliations:** Beijing Key Laboratory of Diabetes Research and Care, Beijing Tongren Hospital, Capital Medical University, No. 1 Dongjiaominxiang Street, Dongcheng District, Beijing 100730, China

## Abstract

Impaired fasting glucose (IFG) is a reversible intermediate hyperglycemia stage with an increasing risk of diabetes and related complications. Our study was designed to identify the relationship between the serum anion gap and the risk of progressing to impaired fasting glucose and diabetes. Here, we performed a prospective, population-based study among 1191 Chinese individuals aged 22–87 years who took health examinations annually between 2006 and 2012 including clinical features and plasma metabolites. All of the participants had no history of diabetes or related chronic complications. Logistic regression analysis was designed to examine the associations between clinical and metabolomic factors and the risk of developing IFG or diabetes. Among them, 58 subjects whose fasting glucose were between 6.1 and 7 mmol/L were diagnosed as IFG or diabetes. After adjusting for age, sex, body mass index (BMI), high-density lipoprotein (HDL), low-density lipoprotein (LDL), alanine aminotransferase (ALT), aspartate aminotransferase (AST), systolic blood pressure (SBP), diastolic blood pressure (DBP), potassium, and albumin at baseline, the participants in the upper tertiles of serum anion gap (SAG) had higher odds of progressing to IFG or diabetes than those in the lower tertiles. A receiver operating characteristic (ROC) curve was analyzed, and the optimal cutoff level for the anion gap to predict incident IFG or diabetes was 13.76 mmol/L, and the area under the ROC curve (AUC) was 0.623. Our data demonstrate that a higher serum anion gap is associated with the risk of developing IFG or diabetes.

## 1. Introduction

Diabetes is actually a collection of metabolic illnesses that usually affect insulin secretion and uptake, with an increase in gluconeogenesis. In 2019, there were approximately 463 million adults aged 20–79 years with diabetes globally, which is projected to increase to 700 million by 2045 [1]. Type 2 diabetes (T2DM) is by far the most common type of diabetes, which imposes a considerable burden on society and patients [2]. Fortunately, T2DM can be prevented or delayed by targeting individuals at high risk [3]. Impaired fasting glucose (IFG) is a prevalent and potentially reversible intermediate stage between normal glucose tolerance and type 2 diabetes [4], and subjects with IFG are at high risk for progression to type 2 diabetes and concomitant complications [5]. Approximately 9% of patients with IFG will develop T2DM without intervention [6]. Identifying the clinical and molecular factors of IFG would enable a reversal, or regression, from IFG to a normoglycemia state, thereby reducing the incidence of diabetes.

A serum anion gap greater than 14 mmol/L is always considered to be abnormally elevated, and a gap of less than 6 mmol/L is considered to be abnormally low [7]. Previous studies showed that an elevated serum anion gap increased the risk for progression to end-stage renal disease. Lower serum bicarbonate is an indication that the SAG is high. We previously showed that lower serum bicarbonate was associated with a higher risk of the development of IFG/DM [8]. Other studies revealed that lower levels of serum bicarbonate and a higher anion gap were associated with insulin resistance [9], but no study has directly measured the effects of SAG on IFG/DM.

Since lower serum bicarbonate reflects higher SAG, we assume that higher SAG may predict the incidence of IFG/DM. Here, we designed a large-scale cohort study to determine the association between a higher serum anion gap and the risk of developing IFG/DM.

## 2. Methods

### 2.1. Study Population

We obtained data for individuals who visited the physical examination center of Beijing Tongren Hospital, Capital Medical University, Beijing, China. Participants were interviewed and underwent physical examinations from 2006 to 2012. A total of 1191 individuals aged 22–87 with fasting plasma glucose (FPG) ranging from 3.9 to 5.5 mmol/L were chosen to take part in the study. Each participant visited the examination center every year for physical and laboratory examinations. All subjects gave informed written consent to participate. Participants with a previous history of cancer, diabetes, thyroid-related disease, history of usage of drugs that could affect acid and blood glucose at baseline or during the observation, and liver, kidney, or other diseases associated with glucose metabolism disorders were excluded. The study was approved by the Human Research Ethics Committee of Beijing Tongren Hospital (No. TRECKY2018-037).

### 2.2. Measurement of Laboratory Parameters

All the individuals who participated in our study received physical examinations, and their morning blood was sampled to ensure that they had fasted for more than 6 hours. Biochemical parameters, including FPG, total cholesterol (TC), triglycerides (TG), low-density lipoprotein cholesterol (LDL-C), high-density lipoprotein cholesterol, serum creatinine (Cr), alanine aminotransferase (ALT), and aspartate aminotransferase (AST), were measured with commercial kits using an automated chemistry analyzer (Beckman Coulter, CA, USA). Serum bicarbonate was measured using an indirect ion-selective electrode method. Weight and height were measured by an MW-900A (Lejia, Hebei, China) and were used to calculate body mass index (BMI) (kg/m^2^). A standard questionnaire was used to evaluate smoking habits, the history of acute and chronic illnesses, and drug use. Blood pressure was measured with the person in a seated position after a 5 min rest with an electronic blood pressure monitor (TM-2656VP, Aieande, Japan). Hypertension was defined as either systolic blood pressure (SBP) ≥140 mm Hg, diastolic blood pressure (DBP) ≥90 mm Hg, or the use of antihypertensive medications. We calculated the serum anion gap according to the following equation: serum anion gap (mmol/L) = serum potassium level (mmol/L) + serum sodium level (mmol/L) - (serum chloride level (mmol/L) + serum bicarbonate level (mmol/L)).

### 2.3. Definition of Progressing to IFG/DM

The American Diabetes Association (ADA) in 1997 defined IFG as FPG ≥ 6.1 mmol/L (≥110 mg/dL) and <7.0 mmol/L (<126 mg/dL) and DM as FPG ≥ 7.0 mmol/L (≥126 mg/dL) [10]. In our study, all of the participants had an FPG from 3.9 to 5.5 mmol/L at baseline and were defined as having IFG/DM with an FPG ≥ 6.1 mmol/L (including ≥7.0 mmol/L) after follow-up.

### 2.4. Statistical Analysis

We stratified the participants into three groups by tertiles (lower, middle, and upper) according to serum anion gap levels at baseline. Clinical categorical variables were reported as frequencies and percentages. Quantitative variables are reported as the means and standard deviations for normally distributed variables or medians and interquartile ranges for nonnormally distributed variables. Comparisons between each group were tested with one-way ANOVA for continuous variables or with the chi-square test for qualitative variables. Three logistic regression models were used to calculate the ORs of progression to IFG/DM. We regard those variables that are possibly related to IFG/DM and SAG levels as potential confounders for adjustment. Model 1 was adjusted for age and sex. Model 2 was adjusted for age, sex, BMI, SBP, DBP, HDL, and LDL. Model 3 was adjusted for age, sex, BMI, SBP, DBP, HDL, LDL, ALT, AST, K+, and albumin. The receiver operating characteristic (ROC) curve of the serum anion gap was conducted for predicting IFG/DM. The optimum cutoff point was defined as the point that had the maximum sensitivity plus specificity. In addition, we calculated the incidence of IFG/DM by quintiles of the distribution of serum anion gap values. We considered a two-sided *a* level of 0.05 for all analyses. All analyses were conducted using the R-4.0.1 software version (http://www.r-project.org).

## 3. Results

### 3.1. Baseline Characteristics of the Study Population

A total of 1191 individuals, including 632 males and 559 females, completed the follow-up and were included in the cohort study. All participants were stratified into three groups based on the tertiles of their SAG levels. The baseline characteristics are shown in Table 1. The SAG levels analyzed ranged from 2.30 to 24.54 mmol/L, with a mean ± SD (13.76 ± 2.96) mmol/L and a median (IQR) of 13.62 (3.82) mmol/L; the normal reference values for serum anion gap were 6–14 mmol/L, and SAG >14.1 mmol/L was considered to be a high level [11]. The participants with higher SAG levels were more likely to be male and younger in age; these participants also had higher weight, BMI, SBP, DBP, ALT, AST, LDL, K^+^, serum Cr and ALB, and lower HDL. After 6 years of follow-up, 58 individuals developed IFG/DM. Each group (serum anion nap low to high) had 10, 16, and 32 IFG/DM (Table 1).

### 3.2. The Relationship between IFG/DM Risk and the SAG by Logistic Regression Analysis

In logistic regression model 1, participants in the upper tertiles of the serum anion gap had higher odds of incident IFG/DM than those in the lower tertiles first (OR: 4.15, 95% CI: 1.95 to 8.83; *P* < 0.001).

In models 2 (OR: 3.77, 95% CI: 1.75 to 8.1; *P* < 0.001) and 3 (OR: 3.38, 95% CI: 1.53 to 7.46; *P* < 0.005), after adjusting for different variables, the ORs were attenuated but remained significant. The participants with serum anion gaps above the median level had higher odds of incident IFG/DM than those below the median (Table 2).

### 3.3. The Value of the SAG for Predicting the Incidence of IFG/DM

**Figure 1:**
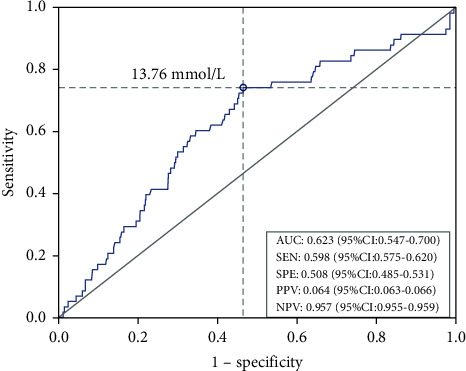
Receiver operating characteristic (ROC) curve of anion gap for predicting impaired fasting glucose (IFG)/diabetes mellitus (DM). The optimal cutoff point for the anion gap was 13.76 mmol/L.

The area under the ROC curve (AUC) of the serum anion gap for predicting IFG/DM was 0.623 (95% CI: 0.547 to 0.700). The analysis of the ROC curve yielded a sensitivity of 59.8%, a specificity of 50.8%, a positive predictive value of 6.4%, and a negative predictive value (NPV) of 95.7%. The optimum cutoff value to predict IFG was 13.76 mmol/L SAG, which indicated that SAG was a predictor for the incidence of IFG/DM (Figure 1).

**Figure 2:**
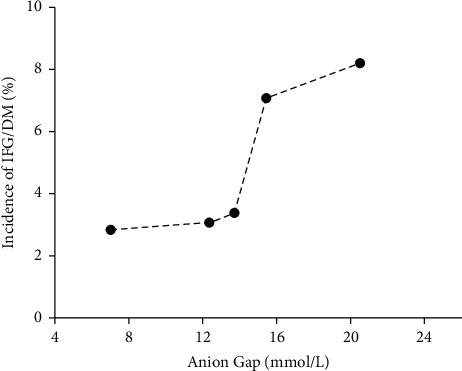
Incidence of impaired fasting glucose (IFG)/diabetes mellitus (DM) by different levels of the baseline serum anion gap.

Next, we analyzed the incidence of IFG/DM by the distribution of SAG values.  Figure 2 shows the incidence of IFG/DM by quintiles of the distribution of serum anion gap values. As the concentration of serum anion gap increased, the percentage of participants progressing to IFG/DM also increased (Figure 2).

## 4. Discussion

In this prospective study, we observed that high levels of SAG increased the incidence of IFG/DM independent of risk factors such as age, sex, BMI, SBP, DBP, HDL, LDL, ALT, AST, and serum Cr, potassium, and albumin. The results of the ROC curve indicated that the SAG level had predictive ability for the development of IFG/DM. In addition, the percentage of participants progressing to IFG/DM increased as SAG increased.

SAG refers to the difference between undetermined anions and cations. It indicates the concentration of fixed acids in plasma, and it is a commonly used and easily obtained laboratory parameter of acid-base imbalance [12]. The elevation of SAG is generally caused by the overproduction of organic acid anions and/or the concomitant and proportionate reduction in the excretion of anions, while changes in the equivalents of total proteins, phosphorus, potassium, and calcium are unusual causes [13]. It has been reported that lactate and ketoanions account for 62% of the increments in SAG [14].

In recent years, many studies have confirmed that elevated SAG is closely associated with poor prognosis in various diseases, including acute pesticide poisoning [15], sepsis [16], acute and chronic kidney injury [17, 18], and coronary artery disease [19]. In a large study, it was shown that increased SAG may be of prognostic significance, as higher levels of AG were associated with hypertension [9].

In our study, the incidence of IFG was higher in men than in women. Individuals with elevated SAG, both men and women, had a high probability of suffering from IFG. Poorer compliance and management in men with diabetes, along with differences in the biological response to hyperglycemia and other risk factors between the sexes [20–22], may explain these findings. Obesity is a strong predictor of an increased risk for type 2 diabetes in adults [23] and probably plays a major role in the development of diabetes [24, 25]. In our study, the participants in the upper tertiles of serum anion gaps had higher levels of weight and BMI, and these findings are consistent with previous studies. Lower HDL and higher LDL were also found in individuals with higher SAG. In recent studies, a high incidence of IFG was significantly and independently associated with low HDL-C levels and increased levels of LDL-C, TC, and TG [26, 27]. Dyslipidemia in this population indicates that obesity can affect insulin secretion or may result in insulin resistance, which may explain this association [26].

Subjects with higher SAG had significantly higher SBP and DBP, and it was found in other studies that in prediabetic hypertensive patients, blood pressure control is less satisfactory than in nondiabetic patients [28, 29]. Furthermore, our study found that ALT and AST were higher as the SAG level increased. Previous studies also indicated a significant association between these parameters and IFG/DM [30, 31] because liver dysfunction associated with chronic hepatitis or liver cirrhosis results in glucose intolerance [32].

Several studies have explored the relationship between SAG and renal diseases. Banerjee et al. suggested that chronic kidney disease (CKD) patients had higher AG which may increase the risk for the progression of end-stage renal disease [18]. Another study showed that AG was associated with an increased risk of 30-day, 90-day, and 365-day all-cause mortality in critically ill patients with acute kidney injury (AKI) [17]. In our study, the serum Cr of the participants was normal, suggesting that the renal function of the patients in our study was normal.

Another factor may be liver function. A previous report showed that there was a weak positive correlation between albumin concentrations and SAG [33]. We evaluated albumin levels in the participants. Although albumin was decreased in our study, the range of albumin was still normal. Thus, the decreased albumin may not affect the results. Furthermore, another study demonstrated that the serum anion gap should be adjusted for low or high serum albumin concentrations [11]. Here, we readjusted for ALT, AST, K+, serum creatinine, and albumin in model 3.

In our study, the AUC of the ROC curve of SAG was 0.623, which suggested that the ability of SAG to discriminate IFG/DM was poor. This may be limited by our sample size. However, the 95.7% NPV results demonstrated great predictive value for the absence of development of IFG/DM. The optimum cutoff value of SAG for predicting progression to IFG/DM was 13.76 mmol/L, which means that SAG above a certain level is harmful. We can see that the optimum cutoff value matches closely with the upper tertiles of the SAG level.

Although the precise mechanism underlying the relationship between SAG and IFG/DM risk has not been fully elucidated, it may be related to insulin resistance, as a previous study has shown that a higher serum anion gap is associated with insulin resistance [9, 34]. Ions play a very important role in maintaining homeostasis and regulating the electrical activities of pancreatic *ß*-cells. The closure of ATP-sensitive potassium channels leads to the depolarization of *ß*-cells and the activation of Ca^2+^ influx, which leads to insulin granule exocytosis and insulin secretion [35, 36]. SAG is related to several ion concentrations, so it may influence the occurrence of IFG through ions. The exact mechanism is still unclear and awaits further investigation and clarification.

However, the present study has three limitations. First, the sample size was small due to the withdrawal of the study halfway, and some participants did not undergo serological examinations. A total of 1314 participants were admitted, but about 150 withdrew or were lost to follow-up. The students who participated in the study were graduated. We tried several adjustments including telephone follow-up and regular workers in the future study. Thus, the number of patients with a final diagnosis of IFG/DM was small, which may have caused deviations in the results. Second, the study population was limited to a single clinical center, raising the possibility that the observed outcomes were specific to this particular patient population. Third, the adjustment for confounding variables influencing glucose metabolism may have been incomplete, such as dietary variables and the consumption of medications that may change the SAG level. These findings need validation in an independent cohort to prove reproducibility.

In conclusion, our present research found an independent association between elevated SAG and a higher risk of progressing to IFG/DM [36]. Controlling SAG at a relatively lower level may aid in the prevention of IFG/DM. Of course, large-scale, multicenter prospective studies are needed to confirm our results. Additional studies are required to explore the underlying mechanism by which the regulation of insulin resistance or insulin secretion could be involved.

## Figures and Tables

**Table 1:** Baseline characteristics of participants by tertiles of serum anion gap.

	By tertiles of serum anion gap			*P* value	Overall
	Low (≤12.51)	Middle (12.52–14.80)	High (≥14.81)		
n	397	397	397		1191
Age (years)	34.6 (9.88)	32.7 (8.18)	30.7 (6.53)	<0.0001	32.6 (8.46)
Sex					
Male	161 (40.6%)	209 (52.6%)	262 (66.0%)	<0.0001	632 (53.1%)
Female	236 (59.4%)	188 (47.4%)	135 (34.0%)		559 (46.9%)
Height (cm)	166 (8.59)	169 (8.72)	170 (8.28)	<0.0001	168 (8.65)
Weight (kg)	64.1 (13.3)	67.4 (13.8)	69.2 (13.9)	<0.0001	66.9 (13.8)
Body mass index (kg/m^2^)	23.0 (3.46)	23.4 (3.56)	23.8 (3.65)	0.0053	23.4 (3.57)
Fasting blood glucose (mmol/L)	5.04 (0.33)	5.04 (0.34)	4.97 (0.34)	0.0016	5.02 (0.34)
Systolic blood pressure (mmHg)	109 (12.5)	112 (12.1)	114 (12.7)	<0.0001	112 (12.6)
Diastolic blood pressure (mmHg)	71.0 (9.07)	73.4 (8.48)	75.6 (8.75)	<0.0001	73.3 (8.96)
ALT (U/L)	21.1 (16.8)	24.4 (17.7)	28.0 (20.6)	<0.0001	24.5 (18.6)
K^+^ (mmol/L)	4.13 (0.273)	4.19 (0.314)	4.23 (0.310)	<0.0001	4.18 (0.302)
Ca^2+^ (mmol/L)	2.37 (1.06)	2.36 (0.0808)	2.38 (0.0792)	0.89	2.37 (0.613)
AST (U/L)	25.3 (8.89)	26.5 (8.11)	28.6 (8.41)	<0.0001	26.8 (8.58)
HDL (mmol/L)	1.48 (0.361)	1.43 (0.367)	1.40 (0.357)	0.015	1.44 (0.363)
LDL (mmol/L)	2.80 (0.688)	2.92 (0.829)	2.99 (0.700)	0.001	2.90 (0.744)
Cr (umol/L)	68.4 (14.7)	71.7 (14.1)	76.0 (14.6)	<0.0001	72.1 (14.8)
Albumin (g/L)	43.4 (2.74)	44.3 (2.71)	45.5 (2.62)	<0.0001	44.4 (2.82)
IFG/DM^*∗*^					
Yes	10 (2.5%)	16 (4.0%)	32 (8.1%)	0.001	58 (4.9%)
No	387 (97.5%)	381 (96.0%)	365 (91.9%)		1133 (95.1%)

Data are *n* (%) or mean (SD). ALT, alanine aminotransferase; AST, aspartate aminotransferase; HDL, high-density lipoprotein; LDL, low-density lipoprotein; Cr, serum creatinine. ^*∗*^Fasting glucose (IFG)/diabetes mellitus (DM).

**Table 2:** Risk of fasting glucose (IFG)/diabetes mellitus (DM) among individuals with a higher serum anion gap, by different adjustment strategies, compared with that of individuals with lower serum anion gap.

Model information	By tertiles of serum anion gap, OR (95% CI)			*P* value^*∗*^
	Low (≤12.51)	Middle (12.52–14.80)	High (≥14.81)	
No. of IFG/DM events (%)	10 (2.5%)	16 (4.0%)	32 (8.1%)	—
Model 1	1.00	1.67 (0.739, 3.75)	4.15 (1.95, 8.83)	0.00023
Model 2	1.00	1.54 (0.681, 3.49)	3.77 (1.75, 8.1)	0.00069
Model 3	1.00	1.50 (0.656, 3.42)	3.38 (1.53, 7.46)	0.0018

^*∗*^ High vs. low. Model 1: adjusted for age and sex. Model 2: model 1+adjusted for body mass index, systolic blood pressure, diastolic blood pressure, high-density lipoprotein (HDL), and low-density lipoprotein (LDL). Model 3: model 2+adjusted for alanine aminotransferase (ALT), aspartate aminotransferase (AST), K^+^, and serum creatinine and albumin.

## Data Availability

All data generated or analyzed during this study are included in this published article.
